# Acute kidney injury among intensive care unit patients in Denmark: temporal changes in mortality and kidney outcomes from 2010 to 2024

**DOI:** 10.1186/s13054-026-06125-3

**Published:** 2026-06-10

**Authors:** Søren Hougaard Christiansen, Henrik Gammelager, Simon Kok Jensen, Christian Fynbo Christiansen

**Affiliations:** 1 Department of Clinical Epidemiology, Aarhus University and Aarhus University Hospital, Olof Palmes Allé 43-45 Aarhus N, 8200 Aarhus, Denmark; 2 Department of Intensive Care, Aarhus University Hospital, Aarhus, Denmark; 3 Department of Clinical Medicine, Aarhus University, Aarhus, Denmark; 4 Department of Anesthesiology and Intensive Care, Aarhus University Hospital, Aarhus, Denmark

**Keywords:** Acute kidney injury, Intensive care unit, Temporal changes, Chronic kidney disease, Kidney failure

## Abstract

**Background:**

The understanding of acute kidney injury (AKI) has evolved, but it remains uncertain whether outcomes have improved over time. This study aimed to describe temporal changes in outcomes among intensive care unit (ICU) patients with AKI in Denmark from 2010 to 2024.

**Methods:**

This cohort study included adult patients with a first-time ICU admission, and AKI identified using creatinine changes in the seven days following ICU admission. The risks of chronic kidney disease (CKD), kidney failure (KF), and death were assessed one year after ICU admission across three periods: 2010–2014, 2015–2019, and 2020–2024. CKD was defined as an outpatient estimated glomerular filtration rate (eGFR) below 60 ml/min/1.73 m2 for more than 90 days. Similarly, KF was defined as an eGFR below 15 ml/min/1.73 m2, a hospital diagnosis, kidney transplantation, or initiation of chronic kidney replacement therapy. Follow-up was divided into seven to 89 days and from 90 to 365 days, and death was considered a competing risk for CKD and KF.

**Results:**

A total of 48,529 patients with AKI were identified. The seven to 89-day mortality was 23.4% (95% confidence interval (CI): 22.5%—24.3%) in 2010–2014, compared to 22.3% (95% CI: 21.7%—22.9%) in 2015–2019, and 20.7% (95% CI: 20.2%—21.2%) in 2020–2024. The 90- to 365-day mortality was 10.3% (95% CI: 9.5%—11.0%) in 2010–2014, 10.2% (95% CI: 9.7%—10.7%) in 2015–2019, and 9.5% (95% CI: 9.1%—10.0%) in 2020–2024. The 90- to 365-day risk of CKD was 21.0% (95% CI: 19.9%—22.1%) in 2010–2014, compared to 19.8% (95% CI: 19.1%—20.6%) in 2015–2019 and 17.3% (95% CI: 16.7%—17.9%) in 2020–2024. The 90- to 365-day risk of KF was 2.2% (95% CI: 1.9%—2.6%) in 2010–2014, 2.1% (95% CI: 1.9%—2.3%) in 2015–2019, and 2.0% (95% CI: 1.8%-2.3%) in 2020–2024.

**Conclusions:**

In this cohort of ICU patients with AKI, mortality and risk of CKD decreased over time, while the risk of KF remained stable. These improvements are encouraging and may reflect changes in ICU management or post-AKI care, although the observational design precludes inference about underlying mechanisms**.**

**Supplementary Information:**

The online version contains supplementary material available at 10.1186/s13054-026-06125-3.

## Introduction

Acute kidney injury (AKI) is a common condition among intensive care unit (ICU) patients and is strongly associated with increased morbidity and mortality [1–4]. The understanding of AKI has evolved from a single-disease entity to a multi-factorial syndrome, characterized by a rapid decrease in kidney function [5, 6]. This has led to the development of consensus guidelines to improve the identification and management of patients with AKI [6]. However, it remains uncertain whether these advancements have translated into improved outcomes over time in ICU patients with AKI. Most studies on temporal changes in prognosis among ICU patients have been restricted to patients receiving kidney replacement therapy (KRT) [7–13], whereas only two studies have included ICU patients with AKI regardless of KRT treatment [14, 15]. Furthermore, existing studies have been restricted to small sample sizes [11, 13, 14], non-population-based settings [8, 11, 13, 14], limited follow-up [8–10, 14, 15], or have relied on AKI diagnosis codes or billing data [7–9, 14, 15]. However, reliance on diagnosis codes and billing data is susceptible to temporal changes in clinical awareness, coding practices, and institutional incentives [16, 17]. Finally, no studies have used longitudinal data to assess temporal changes in the prognosis of chronic kidney disease (CKD), despite AKI being a well-known risk factor [4]. Robust longitudinal data with consistent case ascertainment are needed to determine whether an evolved understanding of AKI has yielded improvements in outcomes in the broader population of ICU patients with AKI. Therefore, we aimed to describe temporal changes in mortality and kidney outcomes among ICU patients with AKI in Denmark from 2010 to 2024.

## Materials and methods

### Study design and setting

We conducted this cohort study utilizing routinely collected administrative and clinical data in Denmark. The Danish healthcare system provides tax-funded universal coverage, including access to both general practitioners and specialized care [18]. Intensive care services are delivered exclusively by public hospitals. Each Danish resident is assigned a unique personal identifier at birth or immigration, enabling individual-level linkage across nationwide registries [18]. The study was registered at Aarhus University (record number 2016–051-000001/812). Ethical approval is not required for non-interventional register-based research in Denmark.

### Study population

Adult patients (aged 18 years or older) admitted to an ICU between January 1, 2010, and December 31, 2024, were identified in the Danish National Patient Registry (DNPR) [19]. The DNPR contains longitudinal data on hospital admissions and diagnoses since 1977, utilizing the 10th version of the International Classification of Diseases (ICD-10) since 1994 (see Additional file 1 with Supplementary Information (SI): Table S1: Codebook) [19]. In addition, the DNPR also contains information on outpatient clinic visits and emergency department contacts since 1995 and hospital-based procedures and treatments since 1999. Admissions to the ICU have been recorded since 2005. To avoid potential bias from recurrent ICU stays, only the first ICU admission per patient during the study period was included. The occurrence of AKI was ascertained within a seven-day window beginning from the time of ICU admission. A schematic overview of the study design, including AKI and covariate assessment and follow-up, is illustrated in SI Figure S1. AKI was defined using the Kidney Disease: Improving Global Outcomes (KDIGO) creatinine criteria: An absolute increase in plasma creatinine of ≥ 26.5 µmol/l within 48 h, a ≥ 1.5-fold increase compared to the lowest creatinine measurement in the preceding seven days, or a ≥ 1.5-fold increase compared to the baseline creatinine [6]. The baseline creatinine used for AKI ascertainment was the median of all creatinine measurements performed in general practice or the outpatient setting within the preceding eight to 365 days. The highest AKI stage obtained within the seven-day ascertainment window was used to categorize the AKI stage. Creatinine data were retrieved from the Clinical Information System Research Database (LABKA) and the Register of Laboratory Results for Research (RLRR), both of which contain information on blood test results from general practitioners and hospitals in Denmark [20, 21]. Since the 1990s, LABKA has expanded its coverage in the Central and Northern regions of Denmark, while RLRR has gained increasing nationwide coverage since the late 2000s. Both registries use the standardized Nomenclature for Properties and Units (NPU) codes [22]. To ensure the availability of preadmission creatinine measurements, the analysis was restricted to patients residing in municipalities with laboratory database coverage for at least two years before ICU admission [22]. This allowed assessment of preexisting kidney function and changes in CKD prevalence over time. Non-Danish residents and patients with preexisting kidney failure (KF) at ICU admission were excluded. KF was defined as at least two outpatient estimated glomerular filtration rate (eGFR) measurements below 15 ml/min/1.73 m^2^ separated by 90 days and with no intervening eGFR measurement above 15 ml/min/1.73 m^2^ during this period. In addition, KF was defined as a hospital diagnosis of KF, chronic KRT, or a history of kidney transplantation [23]. The Chronic Kidney Disease Epidemiology Collaboration (CKD-EPI) 2009 equation, assuming non-black race, was used to calculate the eGFR using outpatient plasma creatinine measurements [24]. 

### Time periods and outcomes

Temporal changes were assessed across three time periods: January 1, 2010, to December 31, 2014 (2010–2014); January 1, 2015, to December 31, 2019 (2015–2019); and January 1, 2020, to December 31, 2024 (2020–2024). Outcomes of interest included CKD, KF, and all-cause mortality. CKD was defined as an outpatient eGFR below 60 ml/min/1.73 m^2^ for more than 90 days and with no intervening eGFR measurement above 60 ml/min/1.73 m^2^ during this period, and was only assessed among patients without preexisting CKD [25]. KF was defined as described above, based on either an eGFR below 15 ml/min/1.73 m^2^ for more than 90 days, a hospital diagnosis, chronic KRT, or kidney transplantation. Information on vital status, emigration, and residency was obtained from the Danish Civil Registration System (CRS), which has had complete nationwide follow-up since 1968 [26].

### Covariates

Information on patient characteristics presented in Table 1 was obtained from the DNPR, the Danish National Prescription Registry (NPR), and the CRS [19, 26, 27]. Treatment with mechanical ventilation, vasopressors, or KRT was defined as initiation of the respective therapy within the first seven days after ICU admission. Admission type was categorized as elective surgical or acute surgical if a surgical procedure had been performed within the seven days preceding ICU admission; all other admissions were classified as non-surgical. Comorbidities were identified using primary and secondary hospital diagnoses recorded during inpatient or outpatient contacts within the 10 years before ICU admission. Hypertension and diabetes were defined as having either a recorded hospital diagnosis or a filled prescription for antihypertensive or antidiabetic medications, respectively. For diabetes, prescriptions for sodium-glucose cotransporter-2 inhibitors or glucagon-like peptide-1 receptor agonists were not considered, as these were increasingly used for non-diabetic indications during the study period. The presence of community-acquired AKI was defined as any creatinine measurement meeting the AKI criteria on the day of hospital admission or during the preceding seven days. The presence of hospital-acquired AKI was defined as any creatinine measurement meeting the AKI criteria from hospital admission to the day of ICU admission. Kidney recovery at day seven was fulfilled if the most recent creatinine measurement was below 120% of baseline creatinine. Preexisting CKD was defined as fulfillment of the CKD definition prior to hospital admission [23]. Among individuals with preexisting CKD, the most recent outpatient eGFR measurement before ICU admission was used to categorize CKD stage. Prehospital medication use was defined as at least one filled prescription within 180 days preceding ICU admission. Prescription data were obtained from the NPR, which contains individual-level records of all prescriptions dispensed from Danish pharmacies since 1994, classified according to the Anatomical Therapeutic Chemical (ATC) system [27]. Information on age, sex, and immigration was obtained from the CRS [26].

**Table 1:** Characteristics of patients with AKI by year of ICU admission

	2010 – 2014 N = 8475 (100%)	2015 – 2019 N = 17,986 (100%)	2020 – 2024 N = 22,068 (100%)
Age (years)	70 (61, 78)	70 (61, 77)	70 (60, 77)
Age categories			
18–39	390 (4.6%)	904 (5.0%)	1205 (5.5%)
40–59	1576 (18.6%)	3345 (18.6%)	4133 (18.7%)
60–79	4828 (57.0%)	10,621 (59.1%)	13,074 (59.2%)
80 +	1681 (19.8%)	3116 (17.3%)	3656 (16.6%)
Male	5059 (59.7%)	11,050 (61.4%)	13,638 (61.8%)
AKI stages			
1	4677 (55.2%)	9639 (53.6%)	11,560 (52.4%)
2	1931 (22.8%)	4145 (23.0%)	5062 (22.9%)
3	1867 (22.0%)	4202 (23.4%)	5446 (24.7%)
Creatinine, baseline (µmol/l)^a^	83 (67, 104)	82 (67, 103)	81 (66, 101)
Creatinine, hospital admission (µmol/l)	97 (73, 146)	99 (74, 148)	98 (74, 147)
Creatinine, ICU admission (µmol/l)	105 (74, 168)	106 (76, 168)	105 (77, 170)
Creatinine, AKI (µmol/l)^b^	153 (108, 238)	159 (114, 247)	162 (117, 253)
KRT	1177 (13.9%)	2464 (13.7%)	2471 (11.2%)
Vasopressor	5028 (59.3%)	11,454 (63.7%)	15,038 (68.1%)
Mechanical ventilation	4946 (58.4%)	10,539 (58.6%)	12,398 (56.2%)
Admission categories			
Acute surgical	2345 (27.7%)	4196 (23.3%)	5183 (23.5%)
Elective surgical	1958 (23.1%)	3066 (17.0%)	3147 (14.3%)
Non-surgical	4172 (49.2%)	10,724 (59.6%)	13,738 (62.3%)
Time from ICU admission to AKI (hours)^c^	41 (15, 96)	34 (13, 84)	34 (13, 74)
Time from hospital admission to ICU (hours)	38 (20, 89)	34 (18, 85)	30 (18, 67)
Community-acquired AKI	2340 (27.6%)	5308 (29.5%)	6287 (28.5%)
Hospital-acquired AKI	2077 (24.5%)	4073 (22.6%)	4734 (21.5%)
Kidney recovery	3745 (44.2%)	7997 (44.5%)	9792 (44.4%)
**Comorbidities**			
CKD^d^	1442 (17.0%)	2764 (15.4%)	3599 (16.3%)
CKD stages			
G3a	518 (6.1%)	1074 (6.0%)	1496 (6.8%)
G3b	582 (6.9%)	1036 (5.8%)	1361 (6.2%)
G4	342 (4.0%)	654 (3.6%)	742 (3.4%)
Hypertension	5927 (69.9%)	12,332 (68.6%)	15,174 (68.8%)
Heart failure	1065 (12.6%)	2467 (13.7%)	2862 (13.0%)
Ischemic heart disease	2195 (25.9%)	3912 (21.8%)	4340 (19.7%)
Atrial fibrillation/flutter	1335 (15.8%)	3141 (17.5%)	3666 (16.6%)
Stroke	746 (8.8%)	1582 (8.8%)	1840 (8.3%)
Peripheral vascular disease	1179 (13.9%)	2471 (13.7%)	3092 (14.0%)
Diabetes	1828 (21.6%)	4293 (23.9%)	4833 (21.9%)
Chronic pulmonary disease	1325 (15.6%)	2935 (16.3%)	3257 (14.8%)
Liver disease	364 (4.3%)	970 (5.4%)	1230 (5.6%)
Cancer	1526 (18.0%)	3430 (19.1%)	4338 (19.7%)
Obstructive urologic disease	703 (8.3%)	1475 (8.2%)	1862 (8.4%)
**Prior medications**			
ACE-I/ARB	3865 (45.6%)	8057 (44.8%)	10,435 (47.3%)
Beta-blocker	2892 (34.1%)	5741 (31.9%)	6806 (30.8%)
Calcium channel blocker	2336 (27.6%)	4497 (25.0%)	6012 (27.2%)
Non-loop diuretic	3046 (35.9%)	5326 (29.6%)	5664 (25.7%)
Loop diuretic	2234 (26.4%)	4330 (24.1%)	4888 (22.1%)
Statin	3329 (39.3%)	6944 (38.6%)	9245 (41.9%)
Antidiabetic	1597 (18.8%)	3739 (20.8%)	5198 (23.6%)
NSAID	1777 (21.0%)	2885 (16.0%)	2846 (12.9%)
SGLT2i	N/A	237 (1.3%)	1629 (7.4%)
GLP1-RA	101 (1.2%)	435 (2.4%)	1301 (5.9%)
**Number of creatinine tests**			
2 years prior to ICU admission	10 (6, 19)	10 (5, 20)	10 (5, 19)
2 years prior to ICU admission, outpatient	4 (2, 8)	4 (2, 9)	5 (2, 9)
From 0 to 6 days	7 (6, 9)	7 (6, 8)	7 (6, 8)
From 7 to 365 days, outpatient	3 (0, 6)	3 (0, 7)	3 (0, 7)

Data are presented as n (%) or median (interquartile range)

^a^ An outpatient baseline creatinine measurement was missing in 15.8% (2010–2014), 15.0% (2015–2019), and 15.5% (2020–2024)

^b^ Creatinine level at the highest AKI stage obtained

^c^ Time from ICU admission to first occurrence of the highest AKI stage

^d^ The prevalence of CKD may be underestimated due to missing outpatient creatinine measurements

AKI: Acute kidney injury, ICU: Intensive care unit, N: Number, KRT: Kidney replacement therapy, CKD: Chronic kidney disease, ACE-I: Angiotensin-converting enzyme inhibitor, ARB: Angiotensin II receptor blocker, NSAID: Non-steroidal anti-inflammatory drug, SGLT2i: Sodium glucose cotransporter 2 inhibitor, GLP1-RA: Glucagon-like peptide 1 receptor agonist

### Statistical analysis

Patient characteristics were tabulated according to the year of the ICU admission (Table 1). Categorical values are reported as frequencies and percentages, while continuous variables are reported as medians with interquartile ranges (IQR). Time-to-event analyses were conducted using day seven after ICU admission as the start of follow-up. Follow-up was divided into two intervals: from seven to 89 days and from 90 to 365 days after ICU admission. For CKD and KF outcomes, follow-up started on day 90. Patients were followed until the first occurrence of the outcome of interest, death, emigration, or end of follow-up 365 days after ICU admission, whichever occurred first. The cumulative incidence (risk) of CKD, KF, and all-cause mortality was estimated. The Aalen-Johansen estimator was used to account for death as a competing risk factor in analyses of CKD and KF. Patients with preexisting CKD were excluded when this outcome was examined. Subgroup analyses were restricted to patients with surgical and non-surgical admissions, those treated with KRT, and those with AKI stage 2 or 3. Additional analyses were conducted among patients residing in the Central and Northern regions of Denmark, where laboratory data coverage was complete throughout the study period, as well as among patients with an available baseline creatinine and preexisting CKD [21]. Analyses were performed using R Studio version 2025.05.1 (R Foundation for Statistical Computing, Vienna, Austria; www.R-project.org).

## Results

**Fig. 1:**
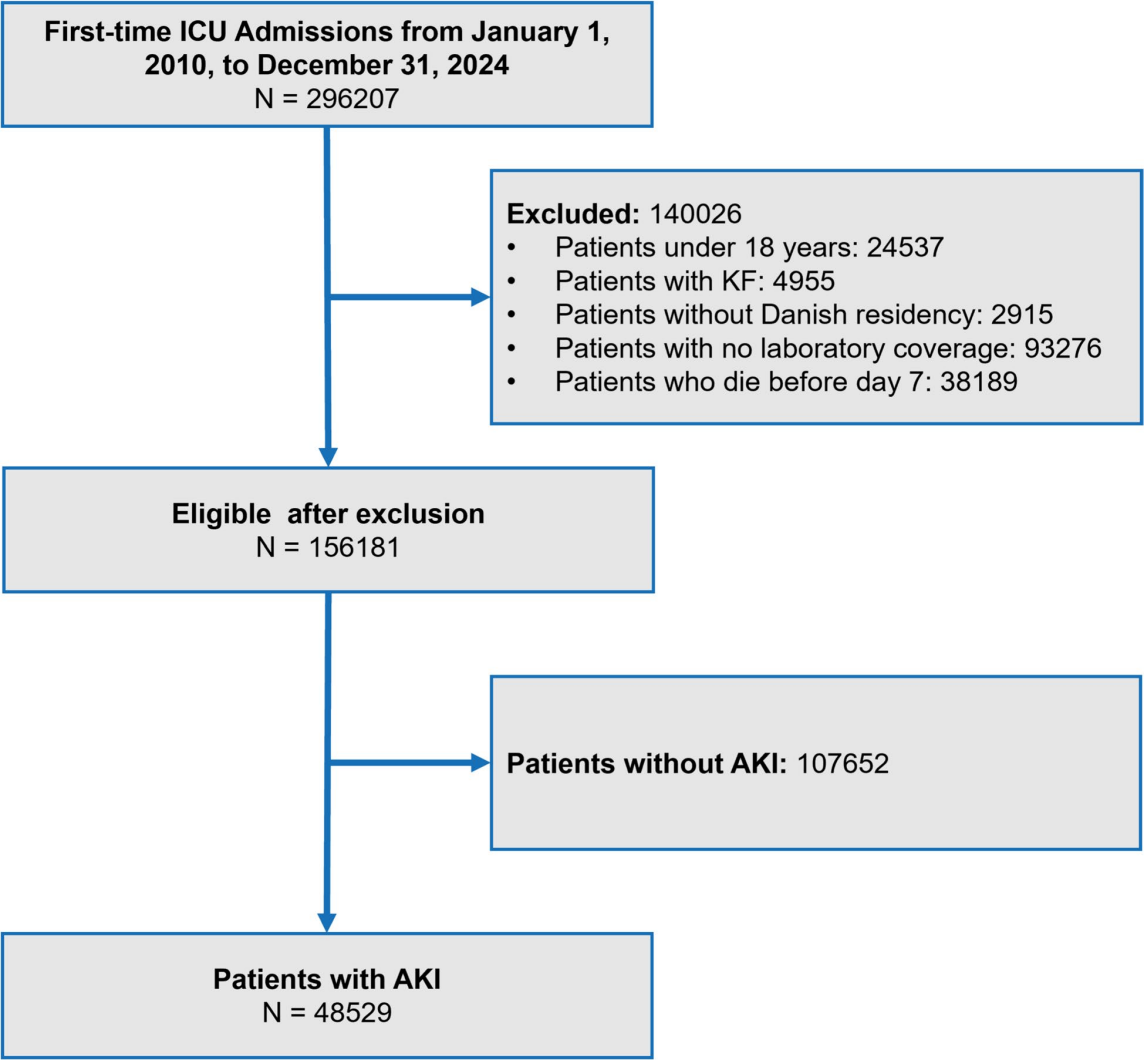
Flowchart of cohort construction. Patients with a first-time ICU admission from January 1, 2010, to December 31, 2024, were identified. Patients were excluded if they were younger than 18 years, had pre-existing KF, were non-residents of Denmark, or lacked laboratory coverage before ICU admission. AKI Acute kidney injury, ICU Intensive care unit; KF Kidney failure; N Number of included patients

A total of 296,207 patients with a first-time ICU admission were identified from January 1, 2010, to December 31, 2024 (Fig. 1). After excluding patients aged below 18 years, those with pre-existing KF, non-Danish residency, individuals with residency in a municipality without laboratory coverage before ICU admission, and those who died before day 7, 156,181 ICU patients remained eligible. Mortality from ICU admission until day 7 was 12.4% (95% confidence interval (CI): 12.1%-12.8%) in 2010–2014, 13.5% (95% CI: 13.2%-13.7%) in 2015–2019, and 13.6% (95% CI: 13.4%-13.9%). Among the eligible patients, 48,529 developed AKI, corresponding to an overall proportion of 31.1%. The proportion of AKI slightly increased over time, from 28.5% (8475 / 29,690) in 2010–2014, compared to 31.7% (17,986 / 56,785) in 2015–2019, and 31.7% (22,068 / 69,706) in 2020–2024. Characteristics of patients with AKI, who were not alive at day seven, are presented in SI: Table S2.

### Patient characteristics

Patient characteristics are presented in Table 1 according to the year of ICU admission. The median age remained stable across the time periods, being 70 (IQR: 61–78) years in 2010–2014, 70 (IQR: 61–77) years in 2015–2019, and 70 (IQR: 60–77) years in 2020–2024. The distribution of AKI stages showed only slight variation over time, with 22.0% in 2010–2014, 23.4% in 2015–2019, and 24.7% in 2020–2024 developing AKI stage 3. The proportion of non-surgical patients increased from 49.2% in 2010–2014 to 59.6% in 2015–2019 and 62.3% in 2020–2024. Most comorbidities remained stable; however, the prevalence of ischemic heart disease declined. Preexisting CKD were stable, with 17.0% affected in 2010–2014, 15.4% in 2015–2019, and 16.3% in 2020–2024.

**Fig. 2:**
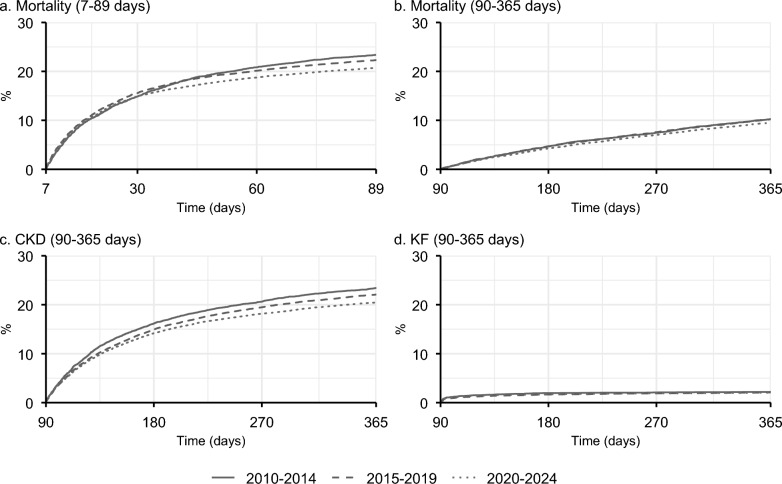
Mortality and risk of CKD and KF by year of ICU admission. **a** and **b** represent mortality from seven to 89 days and 90 to 365 days after ICU admission, respectively. **c** and **d** represent the risks of CKD and KF from day 90 to 365, respectively. CKD Chronic kidney disease, ICU Intensive care unit, KF Kidney failure

### Mortality and risk of CKD and KF

The results of the analyses are presented in Table 2, and Fig. 2. illustrates the risks associated with each outcome. The seven to 89-day mortality decreased from 23.4% (95% CI: 22.5%—24.3%) in 2010–2014 to 22.3% (95% CI: 21.7%—22.9%) in 2015–2019 and further to 20.7% (95% CI: 20.2%—21.2%) in 2020–2024. The 90- to 365-day mortality was 10.3% (95% CI: 9.5%—11.0%) in 2010–2014, 10.2% (95% CI: 9.7%—10.7%) in 2015–2019, and 9.5% (95% CI: 9.1%—10.0%) in 2020–2024. The risk of CKD decreased from 21.0% (95% CI: 19.9%—22.1%) in 2010–2014 to 19.8% (95% CI: 19.1%—20.6%) in 2015–2019 and further decreased to 17.3% (95% CI: 16.7%—17.9%) in 2020–2024. In contrast, the risk of KF remained stable, being 2.2% (95% CI: 1.9%—2.6%) in 2010–2014 compared to 2.1% (95% CI: 1.9%—2.3%) in 2015–2019 and 2.0% (95% CI: 1.8%—2.3%) in 2020–2024.

**Table 2:** Mortality and risk of CKD and KF by year of ICU admission

	2010 – 2014		2015 – 2019		2020 – 2024	
	N	Risk	N	Risk	N	Risk
Mortality (7–89 days)	8475	23.4% (22.5%—24.3%)	17,986	22.3% (21.7%—22.9%)	22,068	20.7% (20.2%—21.2%)
Mortality (90–365 days)	6493	10.3% (9.5%—11.0%)	13,971	10.2% (9.7%—10.7%)	17,124	9.5% (9.1%—10.0%)
CKD (90–365 days)	5488	21.0% (19.9%—22.1%)	12,021	19.8% (19.1%—20.6%)	14,561	17.3% (16.7%—17.9%)
KF (90–365 days)	6493	2.2% (1.9%—2.6%)	13,971	2.1% (1.9%—2.3%)	17,124	2.0% (1.8%—2.3%)

Data are presented as cumulative risks with 95% confidence intervals

N: Number of included patients, CKD: Chronic kidney disease, ICU: Intensive care unit, KF: Kidney failure

**Fig. 3:**
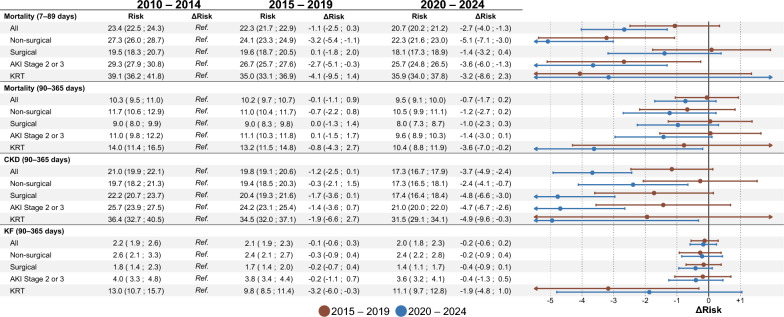
Forest plot of risks and risk differences in outcomes with 95% CIs by year of ICU admission. Risks and risk differences in the complete study population and across subgroups of non-surgical and surgical patients, those with AKI stage 2 or 3, and those treated with KRT. AKI Acute kidney injury, CI Confidence interval, CKD Chronic kidney disease, ICU Intensive care unit, KF Kidney failure, KRT Kidney replacement therapy, Ref Reference, ΔRisk Risk difference

### Subgroup analyses

Results from the subgroup analyses are presented in Fig. 3. Patient characteristics for each subgroup are provided in SI: Table S3-S7. The decrease in seven- to 89-day mortality was consistent among patients with non-surgical admissions, those treated with KRT, and those with AKI stage 2 or 3; however, among surgical patients, the seven- to 89-day mortality remained stable between 2010–2014 and 2015–2019, followed by a decline in 2020–2024. The stable 90- to 365-day mortality observed between 2010–2014 and 2015–2019, followed by a slight decrease in 2020–2024, was consistent across subgroups. The risk of CKD decreased during the study period in all subgroups. The risk of KF was stable among surgical and non-surgical patients and patients with AKI stage 2 or 3; however, it may have decreased among patients treated with KRT. The results of the additional analyses of patients residing in the Northern or Central regions of Denmark (SI: Table S8), patients with an available baseline creatinine (SI: Table S9), and patients with preexisting CKD (SI: Table S10) are similar to those of the primary analysis. 

## Discussion

In this cohort study examining temporal changes in outcomes among adult ICU patients with AKI in Denmark, we observed a decrease in mortality within the first 89 days after ICU admission, while mortality between 90 and 365 days was stable. The risk of CKD decreased, while the risk of KF remained stable.

This is the first study to examine temporal changes in mortality and kidney outcomes beyond hospital discharge in a cohort of ICU patients with laboratory-confirmed AKI. Most existing studies have focused on ICU patients treated with KRT [7–13]. Since only a small fraction of ICU patients with AKI require treatment with KRT [1], results from these studies may not apply to the broader ICU patient population with AKI. Additionally, changes in KRT availability or evolving evidence from randomized controlled trials supporting a watchful waiting approach strategy may have shifted the thresholds for starting treatment over time [4, 15, 16]. These shifts could influence the patient population and lead to temporal changes in outcomes not driven by improvements in treatment or clinical awareness, but by patient selection. In contrast, including patients based on routinely collected laboratory data and not requiring clinicians to initiate treatment actively may be less affected by these shifts in treatment strategies. Other previous studies have relied on either AKI diagnosis codes or billing data to identify the study population with AKI or those treated with KRT [7–9, 14, 15]. However, code-based approaches are susceptible to bias from temporal changes in coding practices, clinical awareness, and potential institutional incentives [16]. These changes may introduce artificial variation in disease occurrence, ascertainment bias, and compromise comparability of study populations across calendar periods. In contrast, a laboratory-based AKI identification using routinely collected serial creatinine measurements may offer a more consistent case ascertainment over time [16]. Finally, the majority of studies have only followed patients until ICU or hospital discharge [8–11, 14, 15]; however, when studying temporal changes, this approach may be susceptible to bias if ICU or hospital discharge patterns evolve.

We observed a decrease in mortality and risk of kidney outcomes over time, which complements and expands upon existing literature. Schmidt et al. examined temporal changes in KRT utilization in ICU stays with AKI and recorded treatment with mechanical ventilation and/or catecholamine infusion from 2008 to 2019 across 34 ICUs in France [15]. Hospital mortality declined from 56.1% to 46.7% over the study period. Individual-level data were not available, AKI was identified through diagnosis codes, and follow-up was limited to hospital discharge. While their findings mirror ours, the more pronounced decrease in mortality may reflect these limitations. Bagshaw et al. studied temporal changes in outcomes among ICU patients with AKI [14]. They included 4,754 patients from 20 Australian ICUs between 1996 and 2005 and defined AKI using a creatinine threshold of ≥ 133 µmol/l, a 24-h urine output < 410 ml, or an AKI diagnosis code in the Acute Physiology and Chronic Health Evaluation at admission. Only 5.2% had AKI, and this proportion increased over time, while in-hospital mortality markedly decreased by 3.4% per year. The use of a fixed creatinine threshold to identify AKI may have led to misclassification of patients with underlying CKD or low baseline creatinine. The increase in the proportion of ICU patients with AKI and decrease in mortality may therefore partly reflect changes in coding practices and case ascertainment. In contrast, our study applied KDIGO AKI creatinine criteria and followed patients for up to one year, enabling more consistent AKI ascertainment and evaluation of outcomes over time. Wald et al. examined temporal changes among 21,234 ICU patients treated with KRT in Ontario, Canada, from 1996 to 2010 using administrative and insurance claims data [7]. Utilization of KRT nearly quadrupled with an increase in annual incidence from 0.8% to 3.0%, while 90-day mortality decreased from 49.9% in 1996–2000 to 45.0% in 2006–2010. Only about 10% of patients had a diagnosis code of AKI. This, alongside the marked annual increase in KRT incidence, underscores the limitations of a code-based approach for identifying KRT treatment and AKI in longitudinal studies.

Some limitations should be considered when interpreting our findings. First, since the study only included patients with AKI, changes in AKI prevention during the study period could have altered the study population. However, if AKI prevention had changed over time, we would also expect a shift in the distribution of AKI stages. Nevertheless, these remained stable across time periods, suggesting limited change in the study population due to prevention and changing severity profile. Secondly, although we restricted the cohort to patients with at least two years of laboratory coverage and used a seven-day AKI ascertainment window, patients presenting with an elevated creatinine without prior baseline measurements may not have been identified with AKI. Similarly, as information on urine output was not available, patients with oliguria may have been misclassified as not having AKI. However, we find it unlikely that misclassification due to oliguria has changed over time. Detection bias due to changes in creatinine testing is also doubtful, as plasma creatinine is routinely measured in ICU patients. The overall incidence of AKI also aligns with prior studies in the ICU population [2]. Third, the risk of CKD may be underestimated due to reduced muscle mass, which lowers creatinine levels and thereby overestimates the eGFR. However, we have no reason to believe that muscle loss differed systematically across time periods. Fourth, during the early part of the study period (i.e., 2010–2014), laboratory data were primarily available for the Central and Northern regions of Denmark, which limited inclusion to residents of these areas. However, Danish regions are generally homogeneous in sociodemographic and healthcare characteristics, and the results from the subgroup analysis were consistent with those from the primary analysis [28]. Finally, we can only speculate about the potential mechanisms underlying the observed decrease in mortality and risk of CKD. Improvements in hemodynamic optimization, fluid management, and the avoidance of nephrotoxic agents may have contributed to improved kidney recovery. The increasing use of sodium-glucose cotransporter 2 inhibitors and glucagon-like peptide 1 receptor agonists, both of which have demonstrated kidney-protective effects, may also play an important role. Changes in case-mix are likely to be important, as we observed an increase in the proportion of non-surgical ICU patients, which may reflect evolving ICU admission practices or improvements in perioperative management. This may also be reflected in the subgroup analyses, in which the decrease in mortality appeared more pronounced among non-surgical than surgical ICU patients. The COVID-19 pandemic may have further contributed to changes in case-mix by altering ICU admission patterns, hospital activity, and introducing a subgroup of patients with a distinct risk profile. Further studies with detailed clinical data are needed to better understand the drivers of these temporal changes.

## Conclusion

In this cohort study of adult ICU patients with AKI in Denmark from 2010 to 2024, mortality within the first 89 days declined over time, whereas mortality between 90 and 365 days remained stable. The risk of CKD decreased, while the risk of KF was stable. While outcomes improved over time, the study design precludes inference about the underlying mechanisms, such as changes in ICU management or post-AKI care.

### Take-home message

In this cohort study of intensive care unit patients with acute kidney injury in Denmark, both mortality and the risk of chronic kidney disease decreased from 2010 to 2024. Further research is needed to clarify whether the improvements may reflect increased clinical awareness and advancements in the management of acute kidney injury.

## Supplementary Information

Additional file 1: Codebook, patient characteristics of subgroups, and results from the sensitivity analysis.

## Data Availability

The data supporting the findings of this study are available from Danish national registries, but are not publicly available due to data protection regulations.

## Abbreviations

AKI: Acute kidney injury
ATC: Anatomical therapeutic chemical
CI: Confidence interval
CRS: Danish civil registration system
CKD: Chronic kidney disease
CKD-EPI: Chronic kidney disease epidemiology collaboration
DNPR: Danish national patient registry
eGFR: Estimated glomerular filtration rate
ICD-10: 10Th version of the international classification of diseases
ICU: Intensive care unit
IQR: Interquartile range
KDIGO: Kidney disease: improving global outcomes
KF: Kidney failure
KRT: Kidney replacement therapy
LABKA: Clinical information system research database
NPR: Danish national prescription registry
NPU: Nomenclature for properties and units
RLRR: Register of laboratory results for research
SI: Supplementary information
