# Supplementary material for: Acute kidney injury among intensive care unit patients in Denmark: temporal changes in mortality and kidney outcomes from 2010 to 2024
Source: Crit Care. 2026 Jun 10;30:419. doi: 10.1186/s13054-026-06125-3 (PMC13474748; doi:10.1186/s13054-026-06125-3)
Supplement: Supplementary file 1 [file 13054_2026_6125_MOESM1_ESM.docx]

SUPPLEMENTARY INFORMATION

Acute kidney injury among intensive care unit patients in Denmark: Temporal changes in mortality and kidney outcomes from 2010 to 2024

**Authors:**

Søren Hougaard Christiansen^1,2,3^, MD; Henrik Gammelager^2,3^, MD, PhD; Simon Kok Jensen^1,3,4^, MD, PhD; Christian Fynbo Christiansen^3^, MD, PhD

**Affiliations:**

^1^Department of Clinical Epidemiology, Aarhus University and Aarhus University Hospital, Aarhus, Denmark

^2^Department of Intensive Care, Aarhus University Hospital, Aarhus, Denmark

^3^Department of Clinical Medicine, Aarhus University, Aarhus, Denmark

^4^Department of Anesthesiology and Intensive Care, Aarhus University Hospital, Aarhus, Denmark

**Corresponding author:**

Søren Hougaard Christiansen; E-mail: shc@clin.au.dk; ORCID: 0000-0001-8124-1022; Address: Olof Palmes Allé 43-45, 8200 Aarhus N, Denmark; Tel.: +45 87167215

**ORCID IDs of co-authors:**

Henrik Gammelager: XXXX; Simon Kok Jensen: 0000-0002-0032-131X; Christian Fynbo Christiansen: 0000-0002-0727-953X

Tables

| **Table S1** Codebook | | | | | |
| --- | --- | --- | --- | --- | --- |
| Variable | Type | Code | Registry | Lookback | Comment |
| Inclusion | | | | | |
| ICU | Procedure code | NABE, NABB | DNPR |  |  |
| AKI | NPU | NPU18016, NPU04998, NPU01807, NPU09101, NPU17559, ASS00355, ASS00356, ASS00354, AAB00327 | RLRR/ LABKA |  |  |
| Exclusion: Kidney failure | | | | | |
| KRT due to CKD | ICD-10 | Z992 | DNPR | 10y |  |
|  | Procedure code | BJFD2 | DNPR | 10y |  |
| CKD stage 5 | ICD | N185 | DNPR | 10y |  |
|  | NPU | See AKI definition for codes | RLRR/LABKA | 2y | Persistent eGFR <15 ml/min/1.73 m² was defined as all eGFR measurements remaining < 15 ml/min/1.73 m² for more than 90 days, with no measurement ≥15 ml/min/1.73 m² occurring during this period |
| Kidney transplant | ICD-10 | Z940, T861 | DNPR | 10y |  |
|  | Surgical code | KKAS | DNPR | 10y |  |
| Outcomes | | | | | |
| CKD | NPU | See AKI definition for codes | RLRR/LABKA |  | Defined as an eGFR persistently < 60 mL/min/1.73 m² for more than 90 days, with no intervening measurement ≥ 60 mL/min/1.73 m² during this period |
| KF |  |  |  |  | See exclusion criteria |
| Death |  | 90 | CRS |  |  |
| Treatments | | | | | |
| KRT | Procedure code | BJFD0 | DNPR |  | Emergency KRT |
| Mechanical ventilation | Procedure code | BGDA0 | DNPR |  |  |
| Vasopressor use | Procedure code | BFHC92, BFHC93, BFHC95 | DNPR |  | Both vasopressors and inotropes |
| Surgery | Surgical code | KA, KB, KC, KD, KE, KG, KH, KJ, KK, KL, KM, KN, KP, KQ, KX, KY, KF | DNPR | 7d | If a patient had both an elective and an acute surgical procedure within the seven days before ICU admission, they were categorized as acute surgical. If no surgical procedure had been performed, they were categorized as non-surgical |
| Community-acquired AKI | NPU | See AKI | RLRR/LABKA | 7d before hospital admission to the day of hospital admission | Any creatinine measurement meeting the AKI criteria at hospital admission and the preceding seven days |
| Hospital-acquired AKI | NPU | See AKI | RLRR/LABKA | Hospital admission to the day of ICU admission | Any creatinine measurement meeting the AKI criteria from hospital admission to the day of ICU admission |
| Kidney recovery | NPU | See AKI | RLRR/LABKA | 7d after ICU admission | Most recent creatinine measurement below 120% of baseline |
| Comorbidities | | | | | |
| CKD | NPU | See outcomes | RLRR | 2y | Baseline kidney function and prevalent CKD were defined based on outpatient creatinine measurements within the two years before ICU admission |
| Hypertension | ICD-10 | I10, I11, I12, I13, I14, I15 | DNPR | 10y | Defined as either a diagnostic code or a prescription of antihypertensive medication |
|  | ATC | C09A, C09B, C09C, C09D | NPR | 180d | ACE-I/ARB |
|  | ATC | C02A, C02B, C02C | NPR | 180d | Alpha blockers |
|  | ATC | C02DA, C02L,  C03A, C03B, C03D, C03E, C03X, C07C, C07D, C08G, C09BA, C09DA,  C09XA52 | NPR | 180d | Non-loop diuretics |
|  | ATC | C02DB, C02DD, C02DG, C04 | NPR | 180d | Vasodilators |
|  | ATC | C07 | NPR | 180d | Beta-blockers |
|  | ATC | C07F, C08, C09BB, C09DB | NPR | 180d | Calcium channel blockers |
| Ischemic heart disease | ICD-10 | I20, I21, I22, I23, DI24, I251, I259 | DNPR | 10y |  |
| Peripheral vascular disease | ICD-10 | I70, I71, I72, I739, I74 | DNPR | 10y |  |
| Heart failure | ICD-10 | I500, I501, I502, I503, I508, I509, I110, I130, I132, I420, I426, I427, I428, I429 | DNPR | 10y |  |
| Atrial fibrillation/flutter | ICD-10 | I48 | DNPR | 10y |  |
| Stroke | ICD-10 | I60, I61, I63, I64 | DNPR | 10y | Hemorrhagic and ischemic stroke |
| Diabetes | ICD-10 | E10, E11, E12, E13, E14, O240, O421, O242, O243, O245, O249, G632, H360, N083 | DNPR | 10y | Defined as either a diagnostic code or a prescription of antidiabetic medication |
|  | ATC | A10A, A10B | NPR | 180d | The following are excluded:  SGLT2i: A10BK, A10BD15, A10BD16, A10BD19, A10BD20, A10BD21, A10BD23, A10BD24, A10BD25, A10BD27  GLP1-RA: A10BJ, A10AE54, A10AE56 |
| Chronic pulmonary disease | ICD-10 | J41, J42, J43, J44, J45, J46 | DNPR | 10y |  |
| Liver disease | ICD-10 | B18, B150, B160, B162, B190, I85, K70, K71, K72, K73, K74,  K760, K766 | DNPR | 10y |  |
| Any cancer not including non-melanoma skin cancer | ICD-10 | C00−26, C30-34, C37-41, C43, C45-58, C60-86, C88, C90-96, C99 | DNPR | 10y |  |
| Urologic obstructive disease | ICD-10 | N13, N20, N21, N22, N40 | DNPR | 10y |  |
| Medications | | | | | |
| ACE-I/ARB | ATC | See hypertension | NPR | 180d |  |
| Beta-blockers | ATC | See hypertension | NPR | 180d |  |
| Calcium channel blockers | ATC | See hypertension | NPR | 180d |  |
| Non-loop diuretics | ATC | See hypertension | NPR | 180d |  |
| Loop diuretics | ATC | C03CA | NPR | 180d |  |
| Statins | ATC | C10AA, C10B | NPR | 180d |  |
| Antidiabetics | ATC | See diabetes | NPR | 180d |  |
| NSAID | ATC | M01A | NPR | 180d |  |
| SGLT2i | ATC | See diabetes | NPR | 180d |  |
| GLP1-RA | ATC | See diabetes | NPR | 180d |  |
| *ICU* Intensive care unit, *DNPR* Danish national patient registry, *AKI* Acute kidney injury, *NPU* Nomenclature for Properties and Units, *RLRR* Register of Laboratory Results for Research, *LABKA* Clinical Information System Research Database, *KF* Kidney failure, *KRT* Kidney replacement therapy, *CKD* Chronic kidney disease, *ICD-10* 10^th^ version of the International Classification of Diseases, *y* years, *eGFR* Estimated glomerular filtration rate, *CRS* Civil registration system, *ATC* Anatomical therapeutic chemical, *NPR* Danish National Prescription Registry, *d* days, *SGLT2i* Sodium-glucose cotransporter-2 inhibitor, *GLP1-RA* Glucagon-like peptide-1 receptor agonist, *ACE-I* Angiotensin-converting enzyme inhibitor, *ARB* Angiotensin II receptor blocker, *NSAID* Non-Steroidal Anti-Inflammatory Drug | | | | | |

| **Table S2** Characteristics of patients with AKI not alive at day 7 by year of ICU admission. | | | |  |
| --- | --- | --- | --- | --- |
|  | **2010 - 2014  N = 2040 (100%)** | **2015 - 2019  N = 4374 (100%)** | **2020 - 2024  N = 5121 (100%)** |  |
| Age (years) | 76 (66, 83) | 74 (66, 82) | 75 (66, 81) |  |
| Age categories |  |  |  |  |
| 18-39 | 31 (1.5%) | 78 (1.8%) | 86 (1.7%) |  |
| 40-59 | 250 (12.3%) | 565 (12.9%) | 659 (12.9%) |  |
| 60-79 | 1043 (51.1%) | 2421 (55.3%) | 2933 (57.3%) |  |
| 80+ | 716 (35.1%) | 1310 (29.9%) | 1443 (28.2%) |  |
| Male | 1122 (55.0%) | 2518 (57.6%) | 3047 (59.5%) |  |
| AKI Stage |  |  |  |  |
| 1 | 803 (39.4%) | 1659 (37.9%) | 1852 (36.2%) |  |
| 2 | 625 (30.6%) | 1303 (29.8%) | 1480 (28.9%) |  |
| 3 | 612 (30.0%) | 1412 (32.3%) | 1789 (34.9%) |  |
| Creatinine, baseline (µmol/l)^a^ | 84 (66, 109) | 82 (66, 105) | 81 (65, 104) |  |
| Creatinine, hospital admission (µmol/l) | 114 (78, 172) | 110 (79, 162) | 109 (77, 160) |  |
| Creatinine, ICU admission (µmol/l) | 126 (85, 195) | 120 (83, 183) | 119 (82, 183) |  |
| Creatinine, AKI (µmol/l)^b^ | 191 (134, 273) | 196 (138, 277) | 199 (139, 290) |  |
| KRT | 288 (14.1%) | 800 (18.3%) | 780 (15.2%) |  |
| Vasopressor | 1351 (66.2%) | 3166 (72.4%) | 3927 (76.7%) |  |
| Mechanical ventilation | 1245 (61.0%) | 2797 (63.9%) | 3164 (61.8%) |  |
| Admission categories |  |  |  |  |
| Acute surgical | 503 (24.7%) | 958 (21.9%) | 1160 (22.7%) |  |
| Elective surgical | 130 (6.4%) | 288 (6.6%) | 313 (6.1%) |  |
| Non-surgical | 1407 (69.0%) | 3128 (71.5%) | 3648 (71.2%) |  |
| Time from ICU admission to AKI (hours)^c^ | 23 (8, 58) | 22 (9, 51) | 22 (9, 48) |  |
| Time from hospital admission to ICU (hours) | 35 (18, 97) | 29 (17, 95) | 28 (17, 82) |  |
| Community-acquired AKI | 794 (38.9%) | 1636 (37.4%) | 1873 (36.6%) |  |
| Hospital-acquired AKI | 634 (31.1%) | 1265 (28.9%) | 1418 (27.7%) |  |
| **Comorbidities** |  |  |  |  |
| CKD^d^ | 474 (23.2%) | 834 (19.1%) | 1045 (20.4%) |  |
| CKD stages |  |  |  |  |
| G3a | 163 (8.0%) | 313 (7.2%) | 429 (8.4%) |  |
| G3b | 182 (8.9%) | 347 (7.9%) | 397 (7.8%) |  |
| G4 | 129 (6.3%) | 174 (4.0%) | 219 (4.3%) |  |
| Hypertension | 1495 (73.3%) | 3109 (71.1%) | 3673 (71.7%) |  |
| Heart failure | 315 (15.4%) | 755 (17.3%) | 805 (15.7%) |  |
| Ischemic heart disease | 525 (25.7%) | 893 (20.4%) | 936 (18.3%) |  |
| Atrial fibrillation/flutter | 411 (20.1%) | 943 (21.6%) | 1149 (22.4%) |  |
| Stroke | 230 (11.3%) | 501 (11.5%) | 595 (11.6%) |  |
| Peripheral vascular disease | 337 (16.5%) | 674 (15.4%) | 706 (13.8%) |  |
| Diabetes | 442 (21.7%) | 998 (22.8%) | 1144 (22.3%) |  |
| Chronic pulmonary disease | 495 (24.3%) | 981 (22.4%) | 1021 (19.9%) |  |
| Liver disease | 147 (7.2%) | 370 (8.5%) | 426 (8.3%) |  |
| Cancer | 455 (22.3%) | 987 (22.6%) | 1196 (23.4%) |  |
| Obstructive urologic disease | 171 (8.4%) | 297 (6.8%) | 365 (7.1%) |  |
| **Prior medications** |  |  |  |  |
| ACE-I/ARB | 859 (42.1%) | 1810 (41.4%) | 2333 (45.6%) |  |
| Beta-blocker | 706 (34.6%) | 1469 (33.6%) | 1680 (32.8%) |  |
| Calcium channel blocker | 535 (26.2%) | 1019 (23.3%) | 1367 (26.7%) |  |
| Non-loop diuretic | 771 (37.8%) | 1279 (29.2%) | 1381 (27.0%) |  |
| Loop diuretic | 783 (38.4%) | 1428 (32.6%) | 1510 (29.5%) |  |
| Statin | 731 (35.8%) | 1615 (36.9%) | 2033 (39.7%) |  |
| Antidiabetic | 375 (18.4%) | 836 (19.1%) | 1134 (22.1%) |  |
| NSAID | 365 (17.9%) | 591 (13.5%) | 569 (11.1%) |  |
| SGLT2i | N/A | 41 (0.9%) | 337 (6.6%) |  |
| GLP1-RA | 13 (0.6%) | 63 (1.4%) | 228 (4.5%) |  |
| **Number of creatinine tests** |  |  |  |  |
| 2 years prior to ICU admission | 13 (7, 25) | 13 (6, 25) | 12 (5, 23) |  |
| 2 years prior to ICU admission, outpatient | 5 (2, 9) | 5 (2, 9) | 5 (2, 10) |  |
| Data are presented as n (%) or median (interquartile range). N/A indicates that the number, median, or a number in the interquartile range is based on five or fewer observations and is therefore masked in accordance with data protection regulations.  ^a^ An outpatient baseline creatinine measurement was missing in 13.7% (2010–2014), 14.5% (2015–2019), and 15.1% (2020–2024)  ^b^ Creatinine level at the highest AKI stage obtained  ^c^ Time from ICU admission to first occurrence of the highest AKI stage  ^d^ The prevalence of CKD may be underestimated due to missing outpatient creatinine measurements  *AKI* Acute kidney injury, *ICU* Intensive care unit, *N* Number, *KRT* Kidney replacement therapy, *CKD* Chronic kidney disease, *ACE-I* Angiotensin-converting enzyme inhibitor, *ARB* Angiotensin II receptor blocker, *NSAID* Non-steroidal anti-inflammatory drug, *SGLT2i* Sodium glucose cotransporter 2 inhibitor, *N/A* Not available, *GLP1-RA* Glucagon-like peptide 1 receptor agonist | | | |  |
|  |  |  |  |  |
|  |  |  |  |  |
|  |  |  |  |  |

| **Table S3** Patient characteristics of patients with AKI stage 2 or 3 by year of ICU admission | | | |  |
| --- | --- | --- | --- | --- |
|  | **2010 - 2014  N = 3798 (100%)** | **2015 - 2019  N = 8347 (100%)** | **2020 - 2024  N = 10508 (100%)** |  |
| Age (years) | 70 (61, 78) | 70 (60, 77) | 70 (60, 77) |  |
| Age categories |  |  |  |  |
| 18-39 | 147 (3.9%) | 374 (4.5%) | 495 (4.7%) |  |
| 40-59 | 714 (18.8%) | 1652 (19.8%) | 2067 (19.7%) |  |
| 60-79 | 2263 (59.6%) | 5094 (61.0%) | 6390 (60.8%) |  |
| 80+ | 674 (17.7%) | 1227 (14.7%) | 1556 (14.8%) |  |
| Male | 2241 (59.0%) | 5046 (60.5%) | 6446 (61.3%) |  |
| AKI Stage |  |  |  |  |
| 2 | 1931 (50.8%) | 4145 (49.7%) | 5062 (48.2%) |  |
| 3 | 1867 (49.2%) | 4202 (50.3%) | 5446 (51.8%) |  |
|  |  |  |  |  |
| Creatinine, baseline (µmol/l)^a^ | 81 (67, 101) | 80 (66, 100) | 80 (65, 99) |  |
| Creatinine, hospital admission (µmol/l) | 106 (76, 193) | 110 (77, 195) | 110 (78, 195) |  |
| Creatinine, ICU admission (µmol/l) | 133 (84, 244) | 133 (86, 240) | 133 (85, 242) |  |
| Creatinine, AKI (µmol/l)^b^ | 236 (165, 353) | 241 (170, 369) | 247 (172, 376) |  |
| KRT | 1047 (27.6%) | 2141 (25.6%) | 2220 (21.1%) |  |
| Vasopressor | 2622 (69.0%) | 5935 (71.1%) | 7818 (74.4%) |  |
| Mechanical ventilation | 2389 (62.9%) | 5103 (61.1%) | 6048 (57.6%) |  |
| Admission categories |  |  |  |  |
| Acute surgical | 1048 (27.6%) | 2002 (24.0%) | 2518 (24.0%) |  |
| Elective surgical | 771 (20.3%) | 1302 (15.6%) | 1331 (12.7%) |  |
| Non-surgical | 1979 (52.1%) | 5043 (60.4%) | 6659 (63.4%) |  |
| Time from ICU admission to AKI (hours)^c^ | 45 (14, 107) | 39 (13, 92) | 40 (14, 85) |  |
| Time from hospital admission to ICU (hours) | 38 (20, 94) | 34 (18, 87) | 30 (18, 72) |  |
| Community-acquired AKI | 1494 (39.3%) | 3484 (41.7%) | 4275 (40.7%) |  |
| Hospital-acquired AKI | 1283 (33.8%) | 2642 (31.7%) | 3142 (29.9%) |  |
| Kidney recovery | 1010 (26.6%) | 2368 (28.4%) | 2996 (28.5%) |  |
| **Comorbidities** |  |  |  |  |
| CKD^d^ | 582 (15.3%) | 1170 (14.0%) | 1578 (15.0%) |  |
| CKD stages |  |  |  |  |
| G3a | 224 (5.9%) | 445 (5.3%) | 645 (6.1%) |  |
| G3b | 216 (5.7%) | 417 (5.0%) | 538 (5.1%) |  |
| G4 | 142 (3.7%) | 308 (3.7%) | 395 (3.8%) |  |
| Hypertension | 2719 (71.6%) | 5765 (69.1%) | 7265 (69.1%) |  |
| Heart failure | 463 (12.2%) | 1102 (13.2%) | 1283 (12.2%) |  |
| Ischemic heart disease | 872 (23.0%) | 1588 (19.0%) | 1788 (17.0%) |  |
| Atrial fibrillation/flutter | 596 (15.7%) | 1396 (16.7%) | 1737 (16.5%) |  |
| Stroke | 297 (7.8%) | 664 (8.0%) | 764 (7.3%) |  |
| Peripheral vascular disease | 542 (14.3%) | 1145 (13.7%) | 1450 (13.8%) |  |
| Diabetes | 885 (23.3%) | 2177 (26.1%) | 2494 (23.7%) |  |
| Chronic pulmonary disease | 532 (14.0%) | 1265 (15.2%) | 1455 (13.8%) |  |
| Liver disease | 186 (4.9%) | 525 (6.3%) | 683 (6.5%) |  |
| Cancer | 716 (18.9%) | 1687 (20.2%) | 2188 (20.8%) |  |
| Obstructive urologic disease | 336 (8.8%) | 687 (8.2%) | 953 (9.1%) |  |
| **Prior medications** |  |  |  |  |
| ACE-I/ARB | 1829 (48.2%) | 3831 (45.9%) | 5116 (48.7%) |  |
| Beta-blocker | 1292 (34.0%) | 2607 (31.2%) | 3204 (30.5%) |  |
| Calcium channel blocker | 1041 (27.4%) | 2076 (24.9%) | 2843 (27.1%) |  |
| Non-loop diuretic | 1431 (37.7%) | 2552 (30.6%) | 2773 (26.4%) |  |
| Loop diuretic | 988 (26.0%) | 2013 (24.1%) | 2273 (21.6%) |  |
| Statin | 1495 (39.4%) | 3198 (38.3%) | 4359 (41.5%) |  |
| Antidiabetic | 775 (20.4%) | 1912 (22.9%) | 2668 (25.4%) |  |
| NSAID | 828 (21.8%) | 1485 (17.8%) | 1513 (14.4%) |  |
| SGLT2i | N/A | 122 (1.5%) | 781 (7.4%) |  |
| GLP1-RA | 55 (1.4%) | 214 (2.6%) | 694 (6.6%) |  |
| **Number of creatinine tests** |  |  |  |  |
| 2 years prior to ICU admission | 11 (6, 20) | 11 (6, 21) | 10 (5, 19) |  |
| 2 years prior to ICU admission, outpatient | 4 (2, 8) | 5 (2, 9) | 5 (2, 9) |  |
| From 0 to 6 days | 8 (7, 9) | 8 (7, 9) | 7 (7, 8) |  |
| From 7 to 365 days, outpatient | 3 (0, 7) | 3 (0, 7) | 3 (0, 7) |  |
| Data are presented as n (%) or median (interquartile range). N/A indicates a number less than 5 and is masked in accordance with data protection regulations.  ^a^ An outpatient baseline creatinine measurement was missing in 14.0% (2010–2014), 12.5% (2015–2019), and 13.8% (2020–2024)  ^b^ Creatinine level at the highest AKI stage obtained  ^c^ Time from ICU admission to first occurrence of the highest AKI stage  ^d^ The prevalence of CKD may be underestimated due to missing outpatient creatinine measurements  *AKI* Acute kidney injury, *ICU* Intensive care unit, *N* Number, *KRT* Kidney replacement therapy, *CKD* Chronic kidney disease, *ACE-I* Angiotensin-converting enzyme inhibitor, *ARB* Angiotensin II receptor blocker, *NSAID* Non-steroidal anti-inflammatory drug, *SGLT2i* Sodium glucose cotransporter 2 inhibitor, *N/A* Not available, *GLP1-RA* Glucagon-like peptide 1 receptor agonist | | | |  |
|  |  |  |  |  |
|  |  |  |  |  |
|  |  |  |  |  |

| **Table S4** Patient characteristics of surgical patients with AKI by year of ICU admission | | | |  |
| --- | --- | --- | --- | --- |
|  | **2010 - 2014  N = 4303 (100%)** | **2015 - 2019  N = 7262 (100%)** | **2020 - 2024  N = 8330 (100%)** |  |
| Age (years) | 71 (63, 79) | 71 (61, 77) | 71 (61, 78) |  |
| Age categories |  |  |  |  |
| 18-39 | 164 (3.8%) | 399 (5.5%) | 502 (6.0%) |  |
| 40-59 | 687 (16.0%) | 1242 (17.1%) | 1373 (16.5%) |  |
| 60-79 | 2595 (60.3%) | 4368 (60.1%) | 5052 (60.6%) |  |
| 80+ | 857 (19.9%) | 1253 (17.3%) | 1403 (16.8%) |  |
| Male | 2687 (62.4%) | 4537 (62.5%) | 5133 (61.6%) |  |
| AKI Stage |  |  |  |  |
| 1 | 2484 (57.7%) | 3958 (54.5%) | 4481 (53.8%) |  |
| 2 | 989 (23.0%) | 1752 (24.1%) | 1925 (23.1%) |  |
| 3 | 830 (19.3%) | 1552 (21.4%) | 1924 (23.1%) |  |
|  |  |  |  |  |
| Creatinine, baseline (µmol/l)^a^ | 84 (69, 103) | 82 (68, 102) | 81 (66, 100) |  |
| Creatinine, hospital admission (µmol/l) | 91 (71, 122) | 92 (72, 125) | 92 (71, 124) |  |
| Creatinine, ICU admission (µmol/l) | 98 (73, 143) | 101 (76, 146) | 100 (75, 147) |  |
| Creatinine, AKI (µmol/l)^b^ | 149 (110, 222) | 157 (115, 234) | 160 (117, 244) |  |
| KRT | 537 (12.5%) | 926 (12.8%) | 861 (10.3%) |  |
| Vasopressor | 2753 (64.0%) | 4958 (68.3%) | 5919 (71.1%) |  |
| Mechanical ventilation | 2789 (64.8%) | 4648 (64.0%) | 5066 (60.8%) |  |
| Admission categories |  |  |  |  |
| Acute surgical | 2345 (54.5%) | 4196 (57.8%) | 5183 (62.2%) |  |
| Elective surgical | 1958 (45.5%) | 3066 (42.2%) | 3147 (37.8%) |  |
| Time from ICU admission to AKI (hours)^b^ | 44 (17, 99) | 36 (14, 87) | 36 (14, 77) |  |
| Time from hospital admission to ICU (hours) | 46 (33, 116) | 42 (24, 110) | 38 (21, 88) |  |
| Community-acquired AKI | 704 (16.4%) | 1430 (19.7%) | 1621 (19.5%) |  |
| Hospital-acquired AKI | 1162 (27.0%) | 1991 (27.4%) | 2080 (25.0%) |  |
| Kidney recovery | 1940 (45.1%) | 3298 (45.4%) | 3726 (44.7%) |  |
| **Comorbidities** |  |  |  |  |
| CKD^d^ | 718 (16.7%) | 1094 (15.1%) | 1400 (16.8%) |  |
| CKD stages |  |  |  |  |
| G3a | 279 (6.5%) | 449 (6.2%) | 622 (7.5%) |  |
| G3b | 290 (6.7%) | 428 (5.9%) | 528 (6.3%) |  |
| G4 | 149 (3.5%) | 217 (3.0%) | 250 (3.0%) |  |
| Hypertension | 3115 (72.4%) | 4951 (68.2%) | 5739 (68.9%) |  |
| Heart failure | 517 (12.0%) | 868 (12.0%) | 845 (10.1%) |  |
| Ischemic heart disease | 1329 (30.9%) | 1708 (23.5%) | 1783 (21.4%) |  |
| Atrial fibrillation/flutter | 658 (15.3%) | 1197 (16.5%) | 1261 (15.1%) |  |
| Stroke | 337 (7.8%) | 570 (7.8%) | 609 (7.3%) |  |
| Peripheral vascular disease | 732 (17.0%) | 1282 (17.7%) | 1533 (18.4%) |  |
| Diabetes | 867 (20.1%) | 1513 (20.8%) | 1559 (18.7%) |  |
| Chronic pulmonary disease | 511 (11.9%) | 890 (12.3%) | 962 (11.5%) |  |
| Liver disease | 128 (3.0%) | 339 (4.7%) | 422 (5.1%) |  |
| Cancer | 949 (22.1%) | 1663 (22.9%) | 1941 (23.3%) |  |
| Obstructive urologic disease | 390 (9.1%) | 618 (8.5%) | 789 (9.5%) |  |
| **Prior medications** |  |  |  |  |
| ACE-I/ARB | 2044 (47.5%) | 3234 (44.5%) | 3924 (47.1%) |  |
| Beta-blocker | 1543 (35.9%) | 2289 (31.5%) | 2462 (29.6%) |  |
| Calcium channel blocker | 1248 (29.0%) | 1843 (25.4%) | 2373 (28.5%) |  |
| Non-loop diuretic | 1584 (36.8%) | 2208 (30.4%) | 2190 (26.3%) |  |
| Loop diuretic | 1017 (23.6%) | 1563 (21.5%) | 1533 (18.4%) |  |
| Statin | 1847 (42.9%) | 2868 (39.5%) | 3601 (43.2%) |  |
| Antidiabetic | 760 (17.7%) | 1312 (18.1%) | 1700 (20.4%) |  |
| NSAID | 907 (21.1%) | 1231 (17.0%) | 1075 (12.9%) |  |
| SGLT2i | N/A | 84 (1.2%) | 530 (6.4%) |  |
| GLP1-RA | 44 (1.0%) | 152 (2.1%) | 449 (5.4%) |  |
| **Number of creatinine tests** |  |  |  |  |
| 2 years prior to ICU admission | 10 (6, 18) | 11 (6, 19) | 10 (5, 18) |  |
| 2 years prior to ICU admission, outpatient | 4 (2, 8) | 5 (2, 8) | 5 (2, 9) |  |
| From 0 to 6 days | 8 (6, 9) | 7 (6, 8) | 7 (6, 8) |  |
| From 7 to 365 days, outpatient | 3 (1, 6) | 3 (1, 7) | 3 (1, 7) |  |
| Data are presented as n (%) or median (interquartile range). N/A indicates a number less than 5 and is masked in accordance with data protection regulations.  ^a^ An outpatient baseline creatinine measurement was missing in 13.9% (2010–2014), 13.8% (2015–2019), and 14.5% (2020–2024)  ^b^ Creatinine level at the highest AKI stage obtained  ^c^ Time from ICU admission to first occurrence of the highest AKI stage  ^d^ The prevalence of CKD may be underestimated due to missing outpatient creatinine measurements *AKI* Acute kidney injury, *ICU* Intensive care unit, *N* Number, *KRT* Kidney replacement therapy, *CKD* Chronic kidney disease, *ACE-I* Angiotensin-converting enzyme inhibitor, *ARB* Angiotensin II receptor blocker, *NSAID* Non-steroidal anti-inflammatory drug, *SGLT2i* Sodium glucose cotransporter 2 inhibitor, *N/A* Not available, *GLP1-RA* Glucagon-like peptide 1 receptor agonist | | | |  |
|  |  |  |  |  |
|  |  |  |  |  |
|  |  |  |  |  |

| **Table S5** Patient characteristics of non-surgical patients with AKI by year of ICU admission | | | |  |
| --- | --- | --- | --- | --- |
|  | **2010 - 2014  N = 4172 (100%)** | **2015 - 2019  N = 10724 (100%)** | **2020 - 2024  N = 13738 (100%)** |  |
| Age (years) | 69 (59, 78) | 70 (60, 77) | 70 (60, 77) |  |
| Age categories |  |  |  |  |
| 18-39 | 226 (5.4%) | 505 (4.7%) | 703 (5.1%) |  |
| 40-59 | 889 (21.3%) | 2103 (19.6%) | 2760 (20.1%) |  |
| 60-79 | 2233 (53.5%) | 6253 (58.3%) | 8022 (58.4%) |  |
| 80+ | 824 (19.8%) | 1863 (17.4%) | 2253 (16.4%) |  |
| Male | 2372 (56.9%) | 6513 (60.7%) | 8505 (61.9%) |  |
| AKI Stage |  |  |  |  |
| 1 | 2193 (52.6%) | 5681 (53.0%) | 7079 (51.5%) |  |
| 2 | 942 (22.6%) | 2393 (22.3%) | 3137 (22.8%) |  |
| 3 | 1037 (24.9%) | 2650 (24.7%) | 3522 (25.6%) |  |
|  |  |  |  |  |
| Creatinine, baseline (µmol/l)^a^ | 81 (65, 105) | 81 (66, 103) | 81 (66, 101) |  |
| Creatinine, hospital admission (µmol/l) | 108 (75, 186) | 105 (75, 170) | 104 (75, 164) |  |
| Creatinine, ICU admission (µmol/l) | 116 (76, 201) | 110 (76, 187) | 110 (77, 187) |  |
| Creatinine, AKI (µmol/l)^b^ | 160 (106, 258) | 161 (112, 259) | 163 (116, 261) |  |
| KRT | 640 (15.3%) | 1538 (14.3%) | 1610 (11.7%) |  |
| Vasopressor | 2275 (54.5%) | 6496 (60.6%) | 9119 (66.4%) |  |
| Mechanical ventilation | 2157 (51.7%) | 5891 (54.9%) | 7332 (53.4%) |  |
| Admission categories |  |  |  |  |
| Non-surgical | 4172 (100.0%) | 10724 (100.0%) | 13738 (100.0%) |  |
| Time from ICU admission to AKI (hours)^c^ | 37 (12, 94) | 32 (12, 82) | 32 (13, 73) |  |
| Time from hospital admission to ICU (hours) | 23 (16, 57) | 25 (16, 61) | 25 (17, 58) |  |
| Community-acquired AKI | 1636 (39.2%) | 3878 (36.2%) | 4666 (34.0%) |  |
| Hospital-acquired AKI | 915 (21.9%) | 2082 (19.4%) | 2654 (19.3%) |  |
| Kidney recovery | 1805 (43.3%) | 4699 (43.8%) | 6066 (44.2%) |  |
| **Comorbidities** |  |  |  |  |
| CKD^d^ | 724 (17.4%) | 1670 (15.6%) | 2199 (16.0%) |  |
| CKD stages |  |  |  |  |
| G3a | 239 (5.7%) | 625 (5.8%) | 874 (6.4%) |  |
| G3b | 292 (7.0%) | 608 (5.7%) | 833 (6.1%) |  |
| G4 | 193 (4.6%) | 437 (4.1%) | 492 (3.6%) |  |
| Hypertension | 2812 (67.4%) | 7381 (68.8%) | 9435 (68.7%) |  |
| Heart failure | 548 (13.1%) | 1599 (14.9%) | 2017 (14.7%) |  |
| Ischemic heart disease | 866 (20.8%) | 2204 (20.6%) | 2557 (18.6%) |  |
| Atrial fibrillation/flutter | 677 (16.2%) | 1944 (18.1%) | 2405 (17.5%) |  |
| Stroke | 409 (9.8%) | 1012 (9.4%) | 1231 (9.0%) |  |
| Peripheral vascular disease | 447 (10.7%) | 1189 (11.1%) | 1559 (11.3%) |  |
| Diabetes | 961 (23.0%) | 2780 (25.9%) | 3274 (23.8%) |  |
| Chronic pulmonary disease | 814 (19.5%) | 2045 (19.1%) | 2295 (16.7%) |  |
| Liver disease | 236 (5.7%) | 631 (5.9%) | 808 (5.9%) |  |
| Cancer | 577 (13.8%) | 1767 (16.5%) | 2397 (17.4%) |  |
| Obstructive urologic disease | 313 (7.5%) | 857 (8.0%) | 1073 (7.8%) |  |
| **Prior medications** |  |  |  |  |
| ACE-I/ARB | 1821 (43.6%) | 4823 (45.0%) | 6511 (47.4%) |  |
| Beta-blocker | 1349 (32.3%) | 3452 (32.2%) | 4344 (31.6%) |  |
| Calcium channel blocker | 1088 (26.1%) | 2654 (24.7%) | 3639 (26.5%) |  |
| Non-loop diuretic | 1462 (35.0%) | 3118 (29.1%) | 3474 (25.3%) |  |
| Loop diuretic | 1217 (29.2%) | 2767 (25.8%) | 3355 (24.4%) |  |
| Statin | 1482 (35.5%) | 4076 (38.0%) | 5644 (41.1%) |  |
| Antidiabetic | 837 (20.1%) | 2427 (22.6%) | 3498 (25.5%) |  |
| NSAID | 870 (20.9%) | 1654 (15.4%) | 1771 (12.9%) |  |
| SGLT2i | N/A | 153 (1.4%) | 1099 (8.0%) |  |
| GLP1-RA | 57 (1.4%) | 283 (2.6%) | 852 (6.2%) |  |
| **Number of creatinine tests** |  |  |  |  |
| 2 years prior to ICU admission | 10 (5, 21) | 10 (5, 21) | 10 (5, 19) |  |
| 2 years prior to ICU admission, outpatient | 4 (2, 8) | 4 (2, 9) | 4 (2, 9) |  |
| From 0 to 6 days | 7 (6, 9) | 7 (6, 8) | 7 (6, 8) |  |
| From 7 to 365 days, outpatient | 2 (0, 6) | 3 (0, 6) | 3 (0, 6) |  |
| Data are presented as n (%) or median (interquartile range). N/A indicates a number less than 5 and is masked in accordance with data protection regulations.  ^a^ An outpatient baseline creatinine measurement was missing in 17.8% (2010–2014), 15.7% (2015–2019), and 16.1% (2020–2024)  ^b^ Creatinine level at the highest AKI stage obtained  ^c^ Time from ICU admission to first occurrence of the highest AKI stage  ^d^ The prevalence of CKD may be underestimated due to missing outpatient creatinine measurements  *AKI* Acute kidney injury, *ICU* Intensive care unit, *N* Number, *KRT* Kidney replacement therapy, *CKD* Chronic kidney disease, *ACE-I* Angiotensin-converting enzyme inhibitor, *ARB* Angiotensin II receptor blocker, *NSAID* Non-steroidal anti-inflammatory drug, *SGLT2i* Sodium glucose cotransporter 2 inhibitor, *N/A* Not available, *GLP1-RA* Glucagon-like peptide 1 receptor agonist | | | |  |
|  |  |  |  |  |
|  |  |  |  |  |
|  |  |  |  |  |

| **Table S6** Patient characteristics of patients with AKI and treated with KRT by year of ICU admission | | | |  |
| --- | --- | --- | --- | --- |
|  | **2010 - 2014  N = 1177 (100%)** | **2015 - 2019  N = 2464 (100%)** | **2020 - 2024  N = 2471 (100%)** |  |
| Age (years) | 69 (60, 76) | 69 (59, 75) | 67 (57, 75) |  |
| Age categories |  |  |  |  |
| 18-39 | 57 (4.8%) | 123 (5.0%) | 167 (6.8%) |  |
| 40-59 | 239 (20.3%) | 543 (22.0%) | 595 (24.1%) |  |
| 60-79 | 735 (62.4%) | 1537 (62.4%) | 1487 (60.2%) |  |
| 80+ | 146 (12.4%) | 261 (10.6%) | 222 (9.0%) |  |
| Male | 738 (62.7%) | 1546 (62.7%) | 1611 (65.2%) |  |
| AKI Stage |  |  |  |  |
| 1 | 130 (11.0%) | 323 (13.1%) | 251 (10.2%) |  |
| 2 | 240 (20.4%) | 492 (20.0%) | 429 (17.4%) |  |
| 3 | 807 (68.6%) | 1649 (66.9%) | 1791 (72.5%) |  |
| Creatinine, baseline (µmol/l)^a^ | 88 (72, 120) | 87 (70, 112) | 86 (69, 108) |  |
| Creatinine, hospital admission (µmol/l) | 130 (88, 274) | 130 (88, 249) | 128 (87, 250) |  |
| Creatinine, ICU admission (µmol/l) | 186 (107, 350) | 172 (102, 333) | 180 (99, 345) |  |
| Creatinine, AKI (µmol/l)^b^ | N/A | N/A | N/A |  |
| KRT | 1177 (100.0%) | 2464 (100.0%) | 2471 (100.0%) |  |
| Vasopressor | 963 (81.8%) | 2002 (81.3%) | 2086 (84.4%) |  |
| Mechanical ventilation | 899 (76.4%) | 1799 (73.0%) | 1755 (71.0%) |  |
| Admission categories |  |  |  |  |
| Acute surgical | 336 (28.5%) | 593 (24.1%) | 592 (24.0%) |  |
| Elective surgical | 201 (17.1%) | 333 (13.5%) | 269 (10.9%) |  |
| Non-surgical | 640 (54.4%) | 1538 (62.4%) | 1610 (65.2%) |  |
| Time from ICU admission to AKI (hours)^c^ | N/A | 52 (16, 120) | 52 (18, 115) |  |
| Time from hospital admission to ICU (hours) | N/A | 34 (18, 97) | 29 (18, 84) |  |
| Community-acquired AKI | 494 (42.0%) | 1058 (42.9%) | 1026 (41.5%) |  |
| Hospital-acquired AKI | 486 (41.3%) | 878 (35.6%) | 837 (33.9%) |  |
| Kidney recovery | 197 (16.7%) | 422 (17.1%) | 361 (14.6%) |  |
| **Comorbidities** |  |  |  |  |
| CKD^d^ | 235 (20.0%) | 416 (16.9%) | 378 (15.3%) |  |
| CKD stages |  |  |  |  |
| G3a | 50 (4.2%) | 123 (5.0%) | 133 (5.4%) |  |
| G3b | 92 (7.8%) | 144 (5.8%) | 133 (5.4%) |  |
| G4 | 93 (7.9%) | 149 (6.0%) | 112 (4.5%) |  |
| Hypertension | 833 (70.8%) | 1643 (66.7%) | 1581 (64.0%) |  |
| Heart failure | 170 (14.4%) | 378 (15.3%) | 315 (12.7%) |  |
| Ischemic heart disease | 278 (23.6%) | 480 (19.5%) | 382 (15.5%) |  |
| Atrial fibrillation/flutter | 184 (15.6%) | 411 (16.7%) | 366 (14.8%) |  |
| Stroke | 90 (7.6%) | 164 (6.7%) | 142 (5.7%) |  |
| Peripheral vascular disease | 187 (15.9%) | 355 (14.4%) | 312 (12.6%) |  |
| Diabetes | 300 (25.5%) | 684 (27.8%) | 608 (24.6%) |  |
| Chronic pulmonary disease | 165 (14.0%) | 339 (13.8%) | 300 (12.1%) |  |
| Liver disease | 66 (5.6%) | 157 (6.4%) | 151 (6.1%) |  |
| Cancer | 211 (17.9%) | 465 (18.9%) | 459 (18.6%) |  |
| Obstructive urologic disease | 103 (8.8%) | 193 (7.8%) | 177 (7.2%) |  |
| **Prior medications** |  |  |  |  |
| ACE-I/ARB | 558 (47.4%) | 1110 (45.0%) | 1122 (45.4%) |  |
| Beta-blocker | 421 (35.8%) | 785 (31.9%) | 700 (28.3%) |  |
| Calcium channel blocker | 334 (28.4%) | 612 (24.8%) | 627 (25.4%) |  |
| Non-loop diuretic | 421 (35.8%) | 732 (29.7%) | 573 (23.2%) |  |
| Loop diuretic | 352 (29.9%) | 641 (26.0%) | 540 (21.9%) |  |
| Statin | 447 (38.0%) | 943 (38.3%) | 972 (39.3%) |  |
| Antidiabetic | 265 (22.5%) | 608 (24.7%) | 667 (27.0%) |  |
| NSAID | 244 (20.7%) | 432 (17.5%) | 368 (14.9%) |  |
| SGLT2i | N/A | 35 (1.4%) | 210 (8.5%) |  |
| GLP1-RA | 17 (1.4%) | 78 (3.2%) | 187 (7.6%) |  |
| **Number of creatinine tests** |  |  |  |  |
| 2 years prior to ICU admission | 11 (5, 22) | 11 (5, 22) | 9 (4, 19) |  |
| 2 years prior to ICU admission, outpatient | 4 (2, 8) | 4 (2, 9) | 4 (2, 9) |  |
| From 0 to 6 days | 8 (7, 10) | 8 (7, 9) | 8 (7, 9) |  |
| From 7 to 365 days, outpatient | 2 (0, 7) | 3 (0, 8) | 3 (0, 8) |  |
| Data are presented as n (%) or median (interquartile range). N/A indicates that the number, median, or a number in the interquartile range is based on five or fewer observations and is therefore masked in accordance with data protection regulations.  ^a^ An outpatient baseline creatinine measurement was missing in 18.4% (2010–2014), 18.3% (2015–2019), and 20.9% (2020–2024)  ^b^ Creatinine level at the highest AKI stage obtained  ^c^ Time from ICU admission to first occurrence of the highest AKI stage  ^d^ The prevalence of CKD may be underestimated due to missing outpatient creatinine measurements *AKI* Acute kidney injury, *ICU* Intensive care unit, *N* Number, *KRT* Kidney replacement therapy, *CKD* Chronic kidney disease, *ACE-I* Angiotensin-converting enzyme inhibitor, *ARB* Angiotensin II receptor blocker, *NSAID* Non-steroidal anti-inflammatory drug, *SGLT2i* Sodium glucose cotransporter 2 inhibitor, *N/A* Not available, *GLP1-RA* Glucagon-like peptide 1 receptor agonist | | | |  |
|  |  |  |  |  |
|  |  |  |  |  |
|  |  |  |  |  |
|  |  |  |  |  |

| **Table S7** Patient characteristics of patients with AKI and residency in Central and Northern Denmark | | | |  |
| --- | --- | --- | --- | --- |
|  | **2010 - 2014  N = 4847 (100%)** | **2015 - 2019  N = 5166 (100%)** | **2020 - 2024  N = 4720 (100%)** |  |
| Age (years) | 71 (61, 79) | 71 (61, 79) | 72 (61, 78) |  |
| Age categories |  |  |  |  |
| 18-39 | 219 (4.5%) | 292 (5.7%) | 284 (6.0%) |  |
| 40-59 | 885 (18.3%) | 910 (17.6%) | 796 (16.9%) |  |
| 60-79 | 2698 (55.7%) | 2902 (56.2%) | 2714 (57.5%) |  |
| 80+ | 1045 (21.6%) | 1062 (20.6%) | 926 (19.6%) |  |
| Male | 2815 (58.1%) | 3034 (58.7%) | 2818 (59.7%) |  |
| AKI Stage |  |  |  |  |
| 1 | 2602 (53.7%) | 2676 (51.8%) | 2418 (51.2%) |  |
| 2 | 1107 (22.8%) | 1203 (23.3%) | 1085 (23.0%) |  |
| 3 | 1138 (23.5%) | 1287 (24.9%) | 1217 (25.8%) |  |
|  |  |  |  |  |
| Creatinine, baseline (µmol/l)^a^ | 83 (67, 104) | 80 (65, 103) | 79 (64, 99) |  |
| Creatinine, hospital admission (µmol/l) | 98 (73, 149) | 101 (73, 156) | 100 (73, 156) |  |
| Creatinine, ICU admission (µmol/l) | 107 (75, 173) | 110 (76, 177) | 110 (76, 184) |  |
| Creatinine, AKI (µmol/l)^b^ | 157 (109, 245) | 159 (111, 256) | 161 (112, 260) |  |
| KRT | 735 (15.2%) | 730 (14.1%) | 490 (10.4%) |  |
| Vasopressor | 2845 (58.7%) | 3271 (63.3%) | 3228 (68.4%) |  |
| Mechanical ventilation | 2644 (54.5%) | 2553 (49.4%) | 2377 (50.4%) |  |
| Admission categories |  |  |  |  |
| Acute surgical | 1284 (26.5%) | 1378 (26.7%) | 1176 (24.9%) |  |
| Elective surgical | 1046 (21.6%) | 763 (14.8%) | 549 (11.6%) |  |
| Non-surgical | 2517 (51.9%) | 3025 (58.6%) | 2995 (63.5%) |  |
| Time from ICU admission to AKI (hours)^c^ | 40 (14, 93) | 35 (13, 89) | 34 (14, 82) |  |
| Time from hospital admission to ICU (hours) | 37 (19, 88) | 27 (17, 70) | 25 (17, 58) |  |
| Community-acquired AKI | 1415 (29.2%) | 1746 (33.8%) | 1560 (33.1%) |  |
| Hospital-acquired AKI | 1203 (24.8%) | 1281 (24.8%) | 1024 (21.7%) |  |
| Kidney recovery | 2160 (44.6%) | 2332 (45.1%) | 2067 (43.8%) |  |
| **Comorbidities** |  |  |  |  |
| CKD^d^ | 815 (16.8%) | 926 (17.9%) | 802 (17.0%) |  |
| CKD stages |  |  |  |  |
| G3a | 297 (6.1%) | 367 (7.1%) | 314 (6.7%) |  |
| G3b | 316 (6.5%) | 343 (6.6%) | 304 (6.4%) |  |
| G4 | 202 (4.2%) | 216 (4.2%) | 184 (3.9%) |  |
| Hypertension | 3383 (69.8%) | 3480 (67.4%) | 3249 (68.8%) |  |
| Heart failure | 671 (13.8%) | 653 (12.6%) | 589 (12.5%) |  |
| Ischemic heart disease | 1251 (25.8%) | 971 (18.8%) | 805 (17.1%) |  |
| Atrial fibrillation/flutter | 771 (15.9%) | 873 (16.9%) | 789 (16.7%) |  |
| Stroke | 461 (9.5%) | 461 (8.9%) | 411 (8.7%) |  |
| Peripheral vascular disease | 706 (14.6%) | 651 (12.6%) | 593 (12.6%) |  |
| Diabetes | 1014 (20.9%) | 1168 (22.6%) | 953 (20.2%) |  |
| Chronic pulmonary disease | 824 (17.0%) | 829 (16.0%) | 639 (13.5%) |  |
| Liver disease | 222 (4.6%) | 255 (4.9%) | 220 (4.7%) |  |
| Cancer | 893 (18.4%) | 1010 (19.6%) | 962 (20.4%) |  |
| Obstructive urologic disease | 401 (8.3%) | 404 (7.8%) | 405 (8.6%) |  |
| **Prior medications** |  |  |  |  |
| ACE-I/ARB | 2189 (45.2%) | 2241 (43.4%) | 2315 (49.0%) |  |
| Beta-blocker | 1613 (33.3%) | 1661 (32.2%) | 1504 (31.9%) |  |
| Calcium channel blocker | 1336 (27.6%) | 1260 (24.4%) | 1308 (27.7%) |  |
| Non-loop diuretic | 1691 (34.9%) | 1498 (29.0%) | 1227 (26.0%) |  |
| Loop diuretic | 1312 (27.1%) | 1308 (25.3%) | 1079 (22.9%) |  |
| Statin | 1916 (39.5%) | 1942 (37.6%) | 2030 (43.0%) |  |
| Antidiabetic | 874 (18.0%) | 1025 (19.8%) | 1060 (22.5%) |  |
| NSAID | 983 (20.3%) | 796 (15.4%) | 560 (11.9%) |  |
| SGLT2i | N/A | 46 (0.9%) | 353 (7.5%) |  |
| GLP1-RA | 59 (1.2%) | 98 (1.9%) | 264 (5.6%) |  |
| **Number of creatinine tests** |  |  |  |  |
| 2 years prior to ICU admission | 11 (6, 20) | 11 (6, 21) | 10 (5, 19) |  |
| 2 years prior to ICU admission, outpatient | 4 (2, 8) | 5 (2, 9) | 5 (2, 9) |  |
| From 0 to 6 days | 8 (6, 9) | 7 (6, 9) | 7 (6, 8) |  |
| From 7 to 365 days, outpatient | 3 (0, 6) | 3 (0, 7) | 3 (0, 7) |  |
| Data are presented as n (%) or median (interquartile range). N/A indicates that the number, median, or a number in the interquartile range is based on five or fewer observations and is therefore masked in accordance with data protection regulations.  ^a^ An outpatient baseline creatinine measurement was missing in 15.5% (2010–2014), 13.7% (2015–2019), and 14.3% (2020–2024)  ^b^ Creatinine level at the highest AKI stage obtained  ^c^ Time from ICU admission to first occurrence of the highest AKI stage  ^d^ The prevalence of CKD may be underestimated due to missing outpatient creatinine measurements  *AKI* Acute kidney injury, *ICU* Intensive care unit, *N* Number, *KRT* Kidney replacement therapy, *CKD* Chronic kidney disease, *ACE-I* Angiotensin-converting enzyme inhibitor, *ARB* Angiotensin II receptor blocker, *NSAID* Non-steroidal anti-inflammatory drug, *SGLT2i* Sodium glucose cotransporter 2 inhibitor, *N/A* Not available, *GLP1-RA* Glucagon-like peptide 1 receptor agonist | | | |  |
|  |  |  |  |  |
|  |  |  |  |  |
|  |  |  |  |  |

| **Table S8** Mortality and risk of CKD and KF among patients with residency in Central and Northern Denmark | | | | | | |
| --- | --- | --- | --- | --- | --- | --- |
|  | **2010 - 2014** | | **2015 - 2019** | | **2020 - 2024** | |
|  | N | Risk | N | Risk | N | Risk |
| Mortality (7-89 days) | 4847 | 24.0% (22.8% - 25.2%) | 5166 | 23.4% (22.2% - 24.5%) | 4720 | 21.5% (20.3% - 22.6%) |
| Mortality (90-365 days) | 3682 | 10.7% (9.7% - 11.7%) | 3957 | 11.0% (10.0% - 12.0%) | 3627 | 11.0% (9.9% - 12.0%) |
| CKD (90-365 days) | 3111 | 21.4% (20.0% - 22.9%) | 3321 | 19.4% (18.1% - 20.8%) | 3044 | 17.7% (16.4% - 19.1%) |
| KF (90-365 days) | 3682 | 2.6% (2.1% - 3.1%) | 3957 | 2.4% (2.0% - 2.9%) | 3627 | 2.3% (1.9% - 2.9%) |
| Data are presented as cumulative risks with 95% confidence intervals.  *N* Number of included patients, *CI* Confidence interval, *CKD* Chronic kidney disease, *ICU* Intensive care unit, *KF* Kidney failure | | | | | | |

| **Table S9** Mortality and risk of CKD and KF among patients with an available baseline creatinine | | | | | | |
| --- | --- | --- | --- | --- | --- | --- |
|  | **2010 - 2014** | | **2015 - 2019** | | **2020 - 2024** | |
|  | N | Risk | N | Risk | N | Risk |
| Mortality (7-89 days) | 7134 | 23.7% (22.7% - 24.7%) | 15294 | 22.5% (21.8% - 23.2%) | 18644 | 21.1% (20.5% - 21.7%) |
| Mortality (90-365 days) | 5441 | 11.0% (10.2% - 11.8%) | 11849 | 10.9% (10.3% - 11.4%) | 14398 | 10.2% ( 9.7% - 10.7%) |
| CKD (90-365 days) | 4456 | 22.5% (21.3% - 23.7%) | 9951 | 21.2% (20.4% - 22.0%) | 11917 | 18.4% (17.8% - 19.2%) |
| KF (90-365 days) | 5441 | 2.4% (2.0% - 2.8%) | 11849 | 2.1% (1.8% - 2.3%) | 14398 | 1.9% (1.7% - 2.2%) |
| Data are presented as cumulative risks with 95% confidence intervals.  *N* Number of included patients, *CI* Confidence interval, *CKD* Chronic kidney disease, *ICU* Intensive care unit, *KF* Kidney failure | | | | | | |

| **Table S10** Mortality, risk of CKD progression, and KF among patients with preexisting CKD | | | | | | |
| --- | --- | --- | --- | --- | --- | --- |
|  | **2010 - 2014** | | **2015 - 2019** | | **2020 - 2024** | |
|  | N | Risk | N | Risk | N | Risk |
| Mortality (7-89 days) | 1442 | 30.3% (27.9% - 32.6%) | 2764 | 29.5% (27.7% - 31.1%) | 3599 | 27.1% (25.7% - 28.6%) |
| Mortality (90-365 days) | 1005 | 14.6% (12.4% - 16.8%) | 1950 | 16.6% (14.9% - 18.3%) | 2563 | 16.1% (14.6% - 17.6%) |
| CKD progression (90-365 days) | 1005 | 27.4% (24.7% - 30.3%) | 1950 | 25.9% (24.1% - 28.0%) | 2563 | 27.5% (25.8% - 29.3%) |
| KF (90-365 days) | 1005 | 5.5% (4.2% - 7.1%) | 1950 | 6.0% (5.0% - 7.2%) | 2563 | 5.7% (4.8% - 6.6%) |
| Data are presented as cumulative risks with 95% confidence intervals.  CKD progression is defined as an increase from the preexisting CKD stage to any higher stage.  *N* Number of included patients, *CI* Confidence interval, *CKD* Chronic kidney disease, *ICU* Intensive care unit, *KF* Kidney failure | | | | | | |

Figures

**Fig. S1** Visualization of covariate assessment and follow-up. *ICU* Intensive care unit, *KF* Kidney failure, *y* years, *d* days, *eGFR* Estimated glomerular filtration rate, *AKI* Acute kidney injury, *CKD* Chronic kidney disease,
